# Overexpression of RIPK4 Predicts Poor Prognosis and Promotes Metastasis in Ovarian Cancer

**DOI:** 10.1155/2021/6622439

**Published:** 2021-06-02

**Authors:** Shaoqiu Liu, Lewei He, Chenchen Sheng, Rongjia Su, Xiaomei Wu, Yunyan Sun, Xiaowei Xi

**Affiliations:** Department of Obstetrics and Gynecology, Shanghai General Hospital, Shanghai Jiao Tong University School of Medicine, Shanghai 200080, China

## Abstract

This study was conducted to evaluate the prognostic value of receptor-interacting protein kinase 4 (RIPK4) in ovarian cancer (OC) and its role in tumorigenesis. RNA expression and the corresponding clinical data were obtained from The Cancer Genome Atlas (TCGA) and Genotype-Tissue Expression (GTEx) databases. The relationship between clinical-pathological characteristics and RIPK4 expression was analyzed using the Wilcoxon signed-rank test and logistic regression. The Cox regression and the Kaplan-Meier method were used to evaluate the relationship between clinicopathological features and overall survival (OS). Gene set enrichment analysis (GSEA) was performed using Molecular Signatures Database. Scratch assay, transwell assay, and cell transfection were used to verify the function of RIPK4. Overexpression of RIPK4 was associated with the stage of OC and distant metastasis. Survival analysis revealed that patients with OC and higher expression of RIPK4 had a poorer prognosis. Univariate and multivariate analyses indicated that high expression of RIPK4 was associated with poor OS, as well as age and stage of OC. The areas under the curve (AUC) at 1, 4, and 8 years were 0.737, 0.634, and 0.669, respectively, according to the established OS prediction model. GSEA revealed that adherens junction, cadherin binding, and Wnt signaling pathway were enriched in the high RIPK4 expression group. Cell transfection confirmed RIPK4 was involved in the Wnt signaling pathway. RIPK4 can act as a potential prognostic molecular marker for poor survival in OC. Moreover, RIPK4 is associated with tumor metastasis and implicated in the regulation of the Wnt signaling pathway.

## 1. Introduction

Ovarian cancer (OC) is the most fatal malignancy of the female reproductive system with multiple histologic subtypes that are characterized by unique metastatic behaviors [1]. The American Cancer Society estimated that there will be approximately 21,750 newly diagnosed ovarian carcinoma cases and more than half of the women will die from ovarian cancer disease in 2020 in the United States [2]. However, the absence of early specific symptoms and effective screening strategies reduces survival above 5 years in a few women after diagnosis [3]. Although the serum level of CA125 is a widely used biomarker, it does not effectively evaluate prognosis and direct treatment [4]. Biomarker research is a major focus of therapeutic studies on OC.

The Wnt/*β*-catenin signaling pathway, as a family of proteins, participates in several vital cellular functions such as stem cell regeneration and organogenesis [5]. Hypo or hyperactivation of the Wnt/*β*-catenin signaling cascade has been associated with human diseases such as cancers [6]. Mutated components of the canonical Wnt signaling are drivers of many cancers [7], including OC [8]. The Wnt/*β*-catenin pathway has been implicated in the pathogenesis of ovarian carcinomas by regulating proliferation, migration, and stemness of ovarian cancer cells [9, 10].

Receptor-interacting protein kinase 4 (RIPK4) is a serine-threonine kinase that is involved in NF-*κ*B and JNK signaling pathways and is processed during apoptosis [11]. RIPK4 knockdown in osteosarcoma can inhibit epithelial-mesenchymal transition by inactivating the Wnt/*β*-catenin signaling pathway [12]. The overexpression of RIPK4 promotes cell migration and invasion in pancreatic cancer [13]. Furthermore, researchers have revealed a simple but effective method of treating bladder cancer using an active small interfering RNA that targets the silencing of RIPK4 expression [14]. Previous studies have revealed that the expression of RIPK4 in ovarian tumor tissues is higher than that of noncancerous ovarian tissues [15]. However, the correlation between RIPK4 and the prognosis of OC is still unclear.

Therefore, the primary aim of this study was to determine the prognostic value of RIPK4 expression in patients with OC based on data acquired from The Cancer Genome Atlas (TCGA) and the Genotype-Tissue Expression (GTEx) databases. Gene set enrichment analysis (GSEA) and in vitro cellular experiments were performed to establish the potential biological pathways involved in ovarian tumorigenesis that are associated with the RIPK4 regulatory network.

The present study demonstrated the adverse impacts of RIPK4 overexpression on the prognosis of human ovarian cancer and the pivotal role of RIPK4 in promoting tumor metastasis. Gene set enrichment analysis and experimental outcomes indicated that the Wnt/*β*-catenin signaling pathway is associated with the high expression level of RIPK4.

## 2. Materials and Methods

### 2.1. Data Acquisition

RNA sequencing data for normal ovarian samples (88 cases) were downloaded from the GTEx database [16]. RNA-Seq data and the corresponding clinical information for patients with ovarian cancer were obtained from TCGA data portal (https://portal.gdc.cancer.gov/) in April 2020. Clinical and demographic data for 587 patients with OC were extracted for further analysis. The inclusion criteria of the study were patients underwent hysterectomy and bilateral adnexectomy and the histology type was serous adenocarcinoma. The patients who underwent other types of surgery or without RNA sequencing dataset were excluded from this study.

### 2.2. Screening for Differentially Expressed Genes

The differentially expressed mRNAs were selected for subsequent analysis using the ∣log_2_ fold change | >2 with an adjusted false discovery rate of *P* < 0.05 and >10^−50^ as the criteria. The volcano and heat map plots were visualized separately using the ggplot2 and pheatmap R packages. The Limma and Beeswarm R packages were used to visualize differential expression of RIPK4 between normal ovarian tissues and ovarian carcinoma tissues.

### 2.3. Enrichment Analysis

GSEA is a statistical algorithm used to determine whether members of a gene set are correlated with the phenotypic class distinction [17]. Each gene set analysis was performed 1000 times, and gene set enrichment analysis was conducted to generate significant prognostic genes with phenotype labels “high-RIPK4” vs. “low-RIPK4” using the Molecular Signatures Database (MSigDB, https://data.broadinstitute.org/gsea/msigdb/collections.jsp). The enriched results considered to be statistically significant in each group were arranged according to nominal *P* value of <0.05 and ∣normalized enrichment score | >1.

### 2.4. Cell Culture

The human OC cell line SKOV3 was obtained from the American Type Culture Collection (ATCC, Manassas, VA, USA). The human OC cell line HO8910 was bought from the Cell Bank of the Chinese Academy of Sciences (Shanghai, China). Both cell lines were maintained in DMEM (Gibco, Thermo Fisher Scientific, Waltham, MA, USA) supplemented with 10% (*v*/*v*) fetal bovine serum (FBS) (Gibco) and 1% (*w*/*v*) penicillin-streptomycin (Gibco), in 5% CO_2_ at 37°C.

### 2.5. Cell Transfection

The small interfering RNA (si-RNA) targeting RIPK4 (si-RIPK4) and the negative control sequence (si-NC) were synthesized by RiboBio (RiboBio, Guangzhou, China). The target sequence for si-RIPK4 was 5′-TCAACGAGGTGGACTTTGA-3′. The nontargeting sequence for si-NC was retained by RiboBio. According to the manufacturer's instructions, the cells were transfected with si-RNA using the Lipofectamine 2000 reagent (Invitrogen, Carlsbad, CA, USA).

### 2.6. Wound Healing Assay

The cells seeded into 6-well plates. When cell confluency reached above 90%, a 200 *μ*l pipette tip was used to make parallel linear scratches. Then, the plates were washed using phosphate-buffered saline (PBS) gently. After that, cells were incubated in serum-free DMEM medium at 37°C for 24 hours. The wound areas were measured under an optical microscope at 0 h and 24 h, respectively.

### 2.7. Transwell Assay

The cells were seeded in serum-free DMEM medium on the upper chamber of transwell inserts (8 *μ*m pore size, Corning, Lowell, MA, USA) uncoated or coated with 30 *μ*g Matrigel (Corning), while the medium on the lower chamber contained 10% FBS. After 24 hours, the nonmigrated or noninvaded cells on the upper surface of the membrane were removed with cotton swabs, while the cells on the lower surface of the membrane were fixed with 4% paraformaldehyde for 15 minutes and then stained with crystal violet staining solution for 15 minutes. The number of the migrated or invaded cells was counted under an inverted microscope.

### 2.8. Western Blot Assay

Total proteins were extracted from the cells using Radio Immunoprecipitation Assay (RIPA) lysis buffer (Beyotime, Shanghai, China) and quantified using the BCA Protein Quantification Kit (Beyotime). The protein lysates were separated by 10% sodium dodecyl sulfate-polyacrylamide gel electrophoresis (SDS-PAGE) (EpiZyme, Shanghai, China) and transferred onto polyvinylidene fluoride (PVDF) membranes (Millipore, Billerica, MA, USA). The membranes were blocked with Tris-buffered saline with 0.1% (*v*/*v*) Tween-20 (TBST) containing 5% (*w*/*v*) nonfat milk for an hour at room temperature and incubated with primary antibodies for RIPK4 (A8495; 1 : 1000; ABclonal Technology, Wuhan, China), *β*-catenin (ab16051; 1 : 1000; Abcam, Cambridge, MA, USA), or *β*-actin (1 : 1000; Proteintech, Chicago, IL, USA) overnight at 4°C. After being washed three times with TBST, the protein bands were incubated with secondary antibodies (1 : 5000; Proteintech) for an hour at room temperature. Finally, the bands were detected by the enhanced chemiluminescence (ECL) Plus kit (Beyotime) and imaged on X-ray film.

### 2.9. Statistical Analysis

R platform (v.3.6.1) was used to perform all statistical analyses. The median value of RIPK4 expression was considered as the cut-off value. The Wilcoxon signed-rank test and logistic regression were used to analyze the correlation between clinical-pathological characteristics and the expression level of RIPK4. The relationship between clinicopathological features and overall survival (OS) was analyzed using Cox regression and the Kaplan-Meier method. The areas under the curve (AUC) at 1, 4, and 8 years were generated from the time-dependent receiver operating characteristic (ROC) curve using the “survivalROC” package. The experimental results were expressed as the mean ± standard deviation (SD). Student's *t*-test or one-way ANOVA was used to analyze the data by the GraphPad Prism V8.0 software (GraphPad Software, USA). *P* < 0.05 was considered to indicate a statistically significant difference. ∗, ∗∗, and ∗∗∗ represented *P* < 0.05, *P* < 0.01, and *P* < 0.001, respectively.

## 3. Results

### 3.1. Differentially Expressed Genes

**Figure 1:**
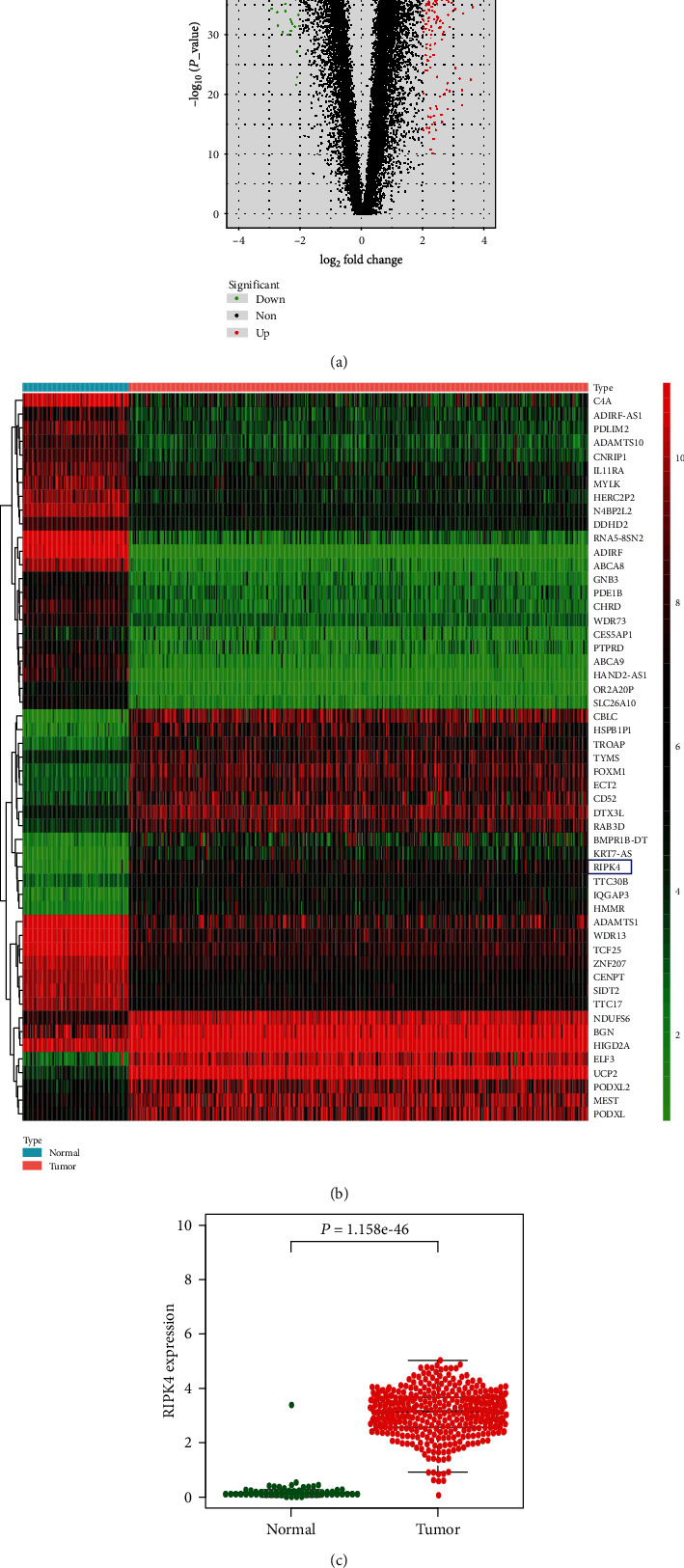
The differentially expressed genes obtained from TCGA and the GTEx databases. (a) Volcano plot representing obtained genes. Red dots denote upregulated genes, green dots denote downregulated genes, and black dots denote nonsignificant genes. (b) Heat map of the top 50 relevant genes selected after screening. (c) Scatter diagram displaying RIPK4 expression between ovarian cancer (red dots) and normal ovarian tissues (green dots) (*P* value < 0.001).

Analysis of differentially expressed genes using R packages revealed that the mRNA expression levels of 1192 genes were downregulated and 1017 genes were upregulated in patients with OC from TCGA database in comparison with normal ovarian samples from the GTEx database (Figure 1(a)). We selected top 50 relevant genes from these differentially expressed genes for further analysis (Figure 1(b)). Excluding the genes that have been reported and studied, we found RIPK4 that is not fully investigated in ovarian carcinoma according to the existing researches. The expression of RIPK4 in ovarian tumor tissues was considerably higher than that in normal ovarian tissues (Figure 1(c)).

### 3.2. Characteristics of Patients with Ovarian Cancer

A total of 587 patients were diagnosed with OC, and the median age of these patients was 59 years (range 26-89 years old). 69 persons of the total patients were of a race other than Asian and White. Except for these 69 people, a proportion of 3.9% of the remaining patients with ovarian tumor were Asian whereas 96.1% were White. All pathological types of the study cohort were serous adenocarcinoma. Owing to 5 out of 587 patients with the absence of clinical stage, the numbers of patients with stage I, II, III, and IV ovarian cancer were 17 (2.9%), 30 (5.2%), 446 (76.6%), and 89 (15.3%), respectively. Distant metastases were detected in 15.3% (*n* = 89) of the ovarian carcinoma cases. Because 7 patients had no follow-up data, a total of 472 among the 580 (81.4%) patients with OC had a survival of less than 5 years and only 18.6% could survive for more than 5 years.

### 3.3. Relationship between RIPK4 Expression and Clinicopathological Features

**Figure 2:**
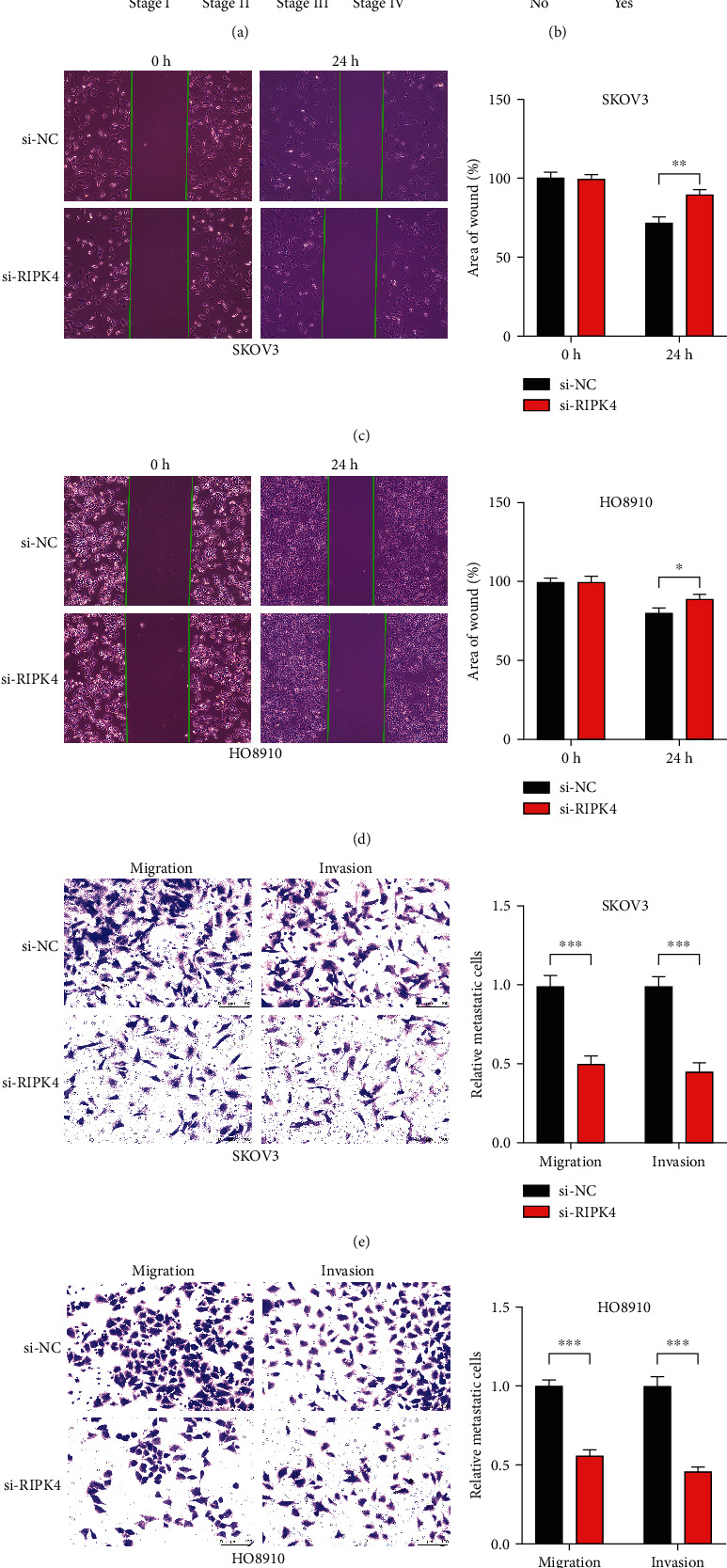
Relationship between RIPK4 expression and clinical-pathological characteristics. (a) Clinical stage. (b) Distant metastasis. (c, d) The wound healing assay in SKOV3 and HO8910 cells after transfection. (e, f) The transwell assay in SKOV3 and HO8910 cells.

The high expression level of RIPK4 was associated with the clinical stage of OC (*P* = 0.023) and distant metastasis (*P* = 0.003) (Figures 2(a) and 2(b)). The patients with higher RIPK4 expression had more aggressive clinical stage and exited distant metastasis lesions more commonly. The logistic regression was used to perform univariate analysis, and results indicated that upregulation of RIPK4 expression was associated with the stage (OR = 3.08 for I+II vs. IV (*P* = 0.026) and OR = 2.08 for III vs. IV (*P* = 0.015)) and distant metastasis (OR = 2.06 for no vs. yes (*P* = 0.016)) in OC (Table 1), while there was no significant relation between the expression of RIPK4 and age and race (*P* > 0.05). The wound healing and transwell migration and invasion assay were performed to detect the role of RIPK4 in OC metastasis. The wound healing assay indicated that the ability of OC cells' migration was weakened after inhibiting RIPK4 expression (Figures 2(c) and 2(d)). The transwell assay results showed that the downregulation of RIPK4 decreased the migration and invasion abilities of SKOV3 and HO8910 cells, compared with the control groups, respectively (Figures 2(e) and 2(f)). These results indicate that increased expression of RIPK4 is a risk factor for the development and metastasis of OC.

### 3.4. Survival Analysis

**Figure 3:**
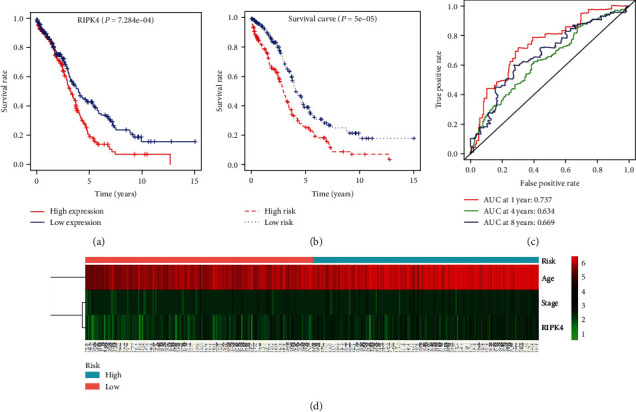
Verification of the efficiency of the prognostic prediction model in patients with ovarian cancer. (a) RIPK4 expression is associated with overall survival in patients with ovarian tumor. (b) The overall survival rate in the high- and low-risk groups according to the prognostic prediction model. (c) The time-dependent receiver operating characteristic curves at different years. (d) A heat map representing the age, stage, and RIPK4 expression for every patient with ovarian tumor based on The Cancer Genome Atlas data.

Patients with OC and low expression of RIPK4 had a better prognosis than patients with high expression of RIPK4 using Cox regression and the Kaplan-Meier method (*P* < 0.001) (Figure 3(a)). The survival rate of 5 years in patients with high RIPK4 expression was 19.7% (95% CI: 13.9%-28.1%), whereas the survival rate in patients with low RIPK4 expression was 42.8% (95% CI: 35.1%-52.0%). Univariate analysis revealed that overexpression of RIPK4 was considerably associated with poor OS (HR = 1.20, 95% CI: 1.01-1.42, *P* = 0.036). Similarly, age (HR = 1.02, 95% CI: 1.01-1.04, *P* = 0.000) and stage (HR = 1.34, 95% CI: 1.00-1.81, *P* = 0.050) were positively correlated with poor OS. Multivariate analysis revealed that RIPK4 was an independent risk factor for OS, with a HR of 1.20 (95% CI: 1.02-1.42, *P* = 0.032), as well as age (HR = 1.03, 95% CI: 1.01-1.04, *P* = 0.000) and stage (HR = 2.50, 95% CI: 1.02-6.14, *P* = 0.045) (Table 2).

### 3.5. Prediction Model for Overall Survival

Results of the survival analysis indicated that age, stage, and RIPK4 expression can serve as independent prognostic factors for unfavorable OS in OC. Therefore, a multivariate Cox model was used to establish the OS prediction model. The risk scores for all patients selected from TCGA database were calculated using the following formula: risk score = age × 0.026 + stage × 0.295 + RIPK4 × 0.182. Survival analysis was performed using a median value (1.02) of the risk scores as the cut-off point for dividing patients into two groups (high-risk and low-risk groups). As shown in Figure 3(b), in the high-risk group, the 5-year survival rate was 26.2% (95% CI: 19.5%-35.1%), whereas in the low-risk group, the 5-year survival rate was 39.1% (95% CI: 31.0%-49.1%) (*P* < 0.001). A time-dependent ROC curve analysis was conducted to test the efficiency of the prediction model. The AUCs at 1-year, 4-year, and 8-year survival rate were 0.737, 0.634, and 0.669, respectively (Figure 3(c)). In addition, the age, stage, and RIPK4 expression of every patient in the high- and low-risk groups were presented in the form of a heat map in Figure 3(d).

### 3.6. Functional Enrichment

**Figure 4:**
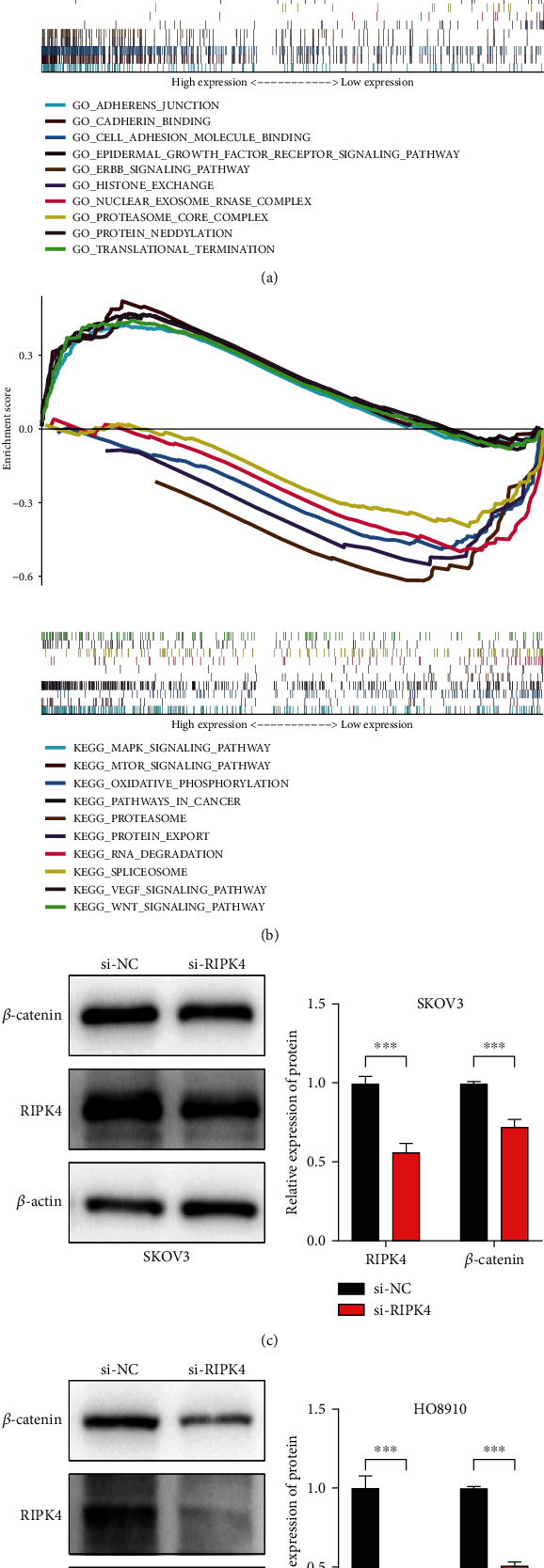
Enrichment results from GSEA. (a) The results of the top 10 relevant gene sets in the GO analysis. (b) The results of the top 10 relevant pathways in the KEGG analysis. (c, d) The protein expression level of RIPK4 and *β*-catenin in SKOV3 and HO8910 cells after transfection.

GSEA was performed using c2 (c2.cp.kegg.v7.1.symbols.gmt) and c5 (c5.all.v7.1.symbols.gmt) with the Molecular Signatures Database gene sets as the reference gene sets, and we focused on the top 10 relevant gene sets in each collection. Citing c5 collection to perform the gene ontology (GO) analysis (Figure 4(a)), it was established that varied expression of RIPK4 was strongly correlated with adherens junction and cadherin binding. The analysis of c2 collection using the Kyoto Encyclopedia of Genes and Genomes (KEGG) database indicated that the Wnt/*β*-catenin signaling pathway was enriched under high expression levels of RIPK4 (Figure 4(b)). In order to verify the result of KEGG analysis, we selected *β*-catenin for further investigation, which is the core molecule of the Wnt/*β*-catenin signaling pathway. As Figures 4(c) and 4(d) show, we used si-RNA to inhibit the expression of RIPK4 and RIPK4 expression was descended efficiently compared with the control group. The western blot assay revealed that decreasing the expression of RIPK4 could negatively affect *β*-catenin expression in SKOV3 and HO8910 cells.

## 4. Discussion

The incidence and mortality of ovarian cancer have reduced over the last few decades, but the deaths caused by OC are substantially higher than the deaths caused by other gynecologic malignancies [3]. Current treatments of OC primarily depend on debulking surgery, platinum-based chemotherapy, antiangiogenic agents, and maintenance therapy of poly (ADP-ribose) polymerase inhibitors. Nonetheless, women suffering from OC still face the challenges of recurrence and drug resistance [18]. Thus, it will be imperative to identify novel biological markers and therapeutic targets that can be used in the treatment of OC.

Studies conducted in the recent past have reported that RIPK4 is involved in the processes of tumor initiation, development, and metastasis. RIPK4 can combine with EZH2, which is a polycomb group of genes that regulates epidermal–mesenchymal transformation and angiogenesis via suppressing tumor suppressor genes, to enhance lymph node metastasis in cervical cancer [19]. Besides, RIPK4 is associated with STAT3 signaling that is activated in tumor metastasis by regulating extracellular matrix remodeling enzymes in lung adenocarcinoma [20]. Moreover, in nasopharyngeal carcinoma, RIPK4 can activate the NF-*κ*B signaling pathway by interacting with the IKK complex to accelerate tumorigenesis and metastasis [21]. It has been reported that RIPK4 plays a prognostic role in a variety of tumors. For example, a study about squamous cell carcinoma of the skin shows that RIPK4 is helpful for the discovery of cancer pathogenesis and for potential treatments [22]. In tongue squamous cell carcinoma, RIPK4 is confirmed to act as a promising biomarker for prognosis and treatment [23]. Furthermore, RIPK4 can also function as a potential diagnostic and independent prognostic biomarker for cervical squamous cell carcinoma [24]. In the case of OC, the expression of RIPK4 was high in tumor tissues and RIPK4 was associated with tumor growth [15]. However, the potential role of RIPK4 in the prognosis of OC still remains unknown. Therefore, the present study was aimed at determining the role of RIPK4 in the prognosis of OC.

The present study indicated that RIPK4 expression in ovarian tumor tissues based on TCGA database RNA sequencing data was remarkably higher than in normal ovarian tissues with reference to data obtained from the GTEx database using bioinformatic analysis. In addition, RIPK4 overexpression was positively correlated with advanced clinical stage, distant metastasis, and poor prognosis of patients with OC. In vitro experiments confirmed that the high expression of RIPK4 was associated with tumor metastasis. The results of GSEA revealed that adherens junction, cadherin binding, and Wnt signaling pathway were enriched in patients with phenotypes of high RIPK4 expression. Meanwhile, it was clearly seen that the expression of *β*-catenin which is known as the core molecule in the Wnt signaling pathway significantly decreased when RIPK4 expression was reduced. This indicates that RIPK4 has a crucial role in the evaluation of development and prognosis of OC.

RIPK4 is a novel member of the receptor-interacting protein kinase family, and its overexpression causes activation of the NF-*κ*B signaling pathway [11]. The upregulation of RIPK4 promotes activation of the NF-*κ*B signaling pathway, upregulates VEGF, and ultimately promotes tumor cell aggressiveness [25]. Conversely, silencing of RIPK4 inhibits the Wnt signaling pathway thus disrupting the pathological process [26]. The present study has demonstrated that upregulation of RIPK4 expression is associated with cellular adhesion and involvement in the Wnt signaling pathways in OC. Nevertheless, a limitation of this study is that we did not elucidate the specific mode of regulation; thus, further studies are required to evaluate the role of RIPK4 in OC.

Notably, we developed a valuable OS assessment model. In this study, we just needed to know the mRNA expression level of RIPK4 and the age and stage of patients with OC and then used the formula, risk score = age × 0.026 + stage × 0.295 + RIPK4 × 0.182, to calculate the risk scores, which were subsequently used to evaluate the prognosis of patients with OC. The prediction model for OS generated by this study will be highly convenient and easily accessible if it is successfully applied in clinical diagnosis and treatment. However, the prediction model has just been verified in this study and the study has some limitations undeniably. More data from multiple cohorts and more evidence from laboratory are needed to verify the prognostic role of RIPK4 in ovarian cancer and the regulatory pathway which RIPK4 is involved in. In addition, it still needs more clinical studies to support the effectiveness of the prediction model for clinical application in the future.

## 5. Conclusions

RIPK4 can function as a potential independent risk factor for overall survival and biomarker in evaluating the prognosis of ovarian cancer. RIPK4 can regulate the Wnt signaling pathway thereby having an effect on ovarian carcinoma. However, further investigations are needed to elucidate the specific molecular mechanisms of RIPK4 in ovarian cancer.

## Figures and Tables

**Table 1:** The relationship between RIPK4 expression^a^ and clinicopathological characteristics.

Clinical characteristics	Total (*N*)	OR (95% CI)	*P* value
Age (continuous)	587	0.99 (0.98-1.01)	0.536
Race (Asian vs. White)	518	0.71 (0.21-2.26)	0.558
Stage			
(I+II vs. IV)	136	3.08 (1.17-8.57)	**0.026**
(III vs. IV)	535	2.08 (1.16-3.80)	**0.015**
Distant metastasis (no vs. yes)	582	2.06 (1.16-3.76)	**0.016**

^a^Categorical dependent variable, more or less than the median value of 3.15.

**Table 2:** Univariate and multivariate analyses representing the correlation between clinicopathological characteristics and overall survival in patients with ovarian cancer.

Clinicopathological variable	Univariate analysis			Multivariate analysis		
	HR	95% CI	*P* value	HR	95% CI	*P* value
Age	1.02	1.01-1.04	**0.000**	1.03	1.01-1.04	**0.000**
Race	0.73	0.27-1.98	0.532	0.49	0.18-1.36	0.174
Stage	1.34	1.00-1.81	**0.050**	2.50	1.02-6.14	**0.045**
Metastasis	1.24	0.87-1.76	0.239	0.47	0.17-1.26	0.133
RIPK4	1.20	1.01-1.42	**0.036**	1.20	1.02-1.42	**0.032**

## Data Availability

Data can be obtained by contacting the corresponding author.
